# Validation of a Portable Tool for Conjunctival Ultraviolet Autofluorescence Imaging

**DOI:** 10.1007/s10439-026-03992-3

**Published:** 2026-01-28

**Authors:** Priya Bahra, Lea Damnjanovic, Koko Faen, Ashish Agar, S. Mojtaba Golzan, Minas T. Coroneo

**Affiliations:** 1 School of Clinical Medicine, Faculty of Medicine and Health, University of New South Wales, Sydney, Australia; 2 Department of Ophthalmology, Prince of Wales Hospital, Sydney, Australia; 3 Vision Science Group, Graduate School of Health (Orthoptics Discipline), University of Technology Sydney, 15 Broadway, Ultimo, NSW 2007 Australia

**Keywords:** Conjunctival ultraviolet autofluorescence (CUVAF), UV exposure, Ocular surface disorders, Portable diagnostics

## Abstract

**Purpose:**

Conjunctival ultraviolet autofluorescence (CUVAF) is an objective biomarker of ocular sun exposure, aiding in the assessment of ophthalmohelioses and other UV-related eye conditions. However, conventional CUVAF imaging requires a bulky and costly benchtop digital single-lens reflex (DSLR) camera system. This study evaluates the validity of a smartphone-based clip-on tool as a portable, affordable alternative for CUVAF imaging.

**Methods:**

95 participants were recruited from an ophthalmology clinic in Sydney, Australia. Nasal and temporal conjunctival images were captured using both DSLR-based and smartphone-based systems. CUVAF was quantified using two parameters: area ratio (CUVAF area relative to visible sclera) and intensity ratio (CUVAF mean intensity relative to adjacent background). Agreement between systems were assessed using Bland–Altman analysis.

**Results:**

The mean age of participants was 58 ± 14 years, with 42% identifying as male. CUVAF area ratios were slightly higher in smartphone images (0.037 ± 0.03) compared to DSLR (0.022 ± 0.02, *p* < 0.01). Intensity ratios showed no significant difference between systems (smartphone: 1.35 ± 0.24, DSLR: 1.37 ± 0.28, *p* = 0.43). Bland–Altman plots demonstrated good agreement, with mean differences of − 0.014 for area and 0.041 for intensity.

**Conclusions:**

The smartphone-based CUVAF imaging tool demonstrates strong agreement with the gold-standard DSLR system, supporting its potential for clinical and field-based assessment of ocular UV exposure. Slight differences in measured CUVAF area are likely attributable to inherent differences in optical geometry and magnification between the two devices, not underlying biological variation.

**Supplementary Information:**

The online version contains supplementary material available at 10.1007/s10439-026-03992-3.

## Introduction

The eye is vulnerable to environmental dangers both by being exposed at a vantage point, in order to subserve its function [1] and by possessing lenticular systems that can focus light [1, 2]. The ocular surface is thus perfectly placed as a sampling site for chronic exposure of both the eye and the skin to potentially damaging wavelengths of the electromagnetic spectrum.

Ultraviolet (UV) radiation is a significant environmental risk factor for various ocular conditions, including pterygium, pinguecula and cataract, collectively known as the ophthalmohelioses [2]. These lesions are particularly prevalent in regions with high UV intensity, such as the “pterygium belt” between 40° North and South latitude but also including arctic environments with high terrain reflectivity. In Australia, despite extensive sun safety campaigns, the burden of UV-related eye diseases remains substantial, especially among rural and Indigenous populations [3, 4].

Accurately quantifying cumulative sun exposure is crucial for both clinical assessment and public health research [5]. Traditional methods, like self-reported time spent outdoors, are susceptible to recall bias and variability [6]. Conjunctival ultraviolet autofluorescence (CUVAF) imaging offers an objective measure of ocular UV exposure [7–9]. This technique captures autofluorescent areas of the conjunctiva using ultraviolet fluorescence photography (UVFP), which are thought to result from subclinical UV-induced cellular changes. Although the exact fluorophores responsible for this autofluorescence are not fully identified, candidates include pyridinoline crosslinks in collagen, tryptophan, and reduced nicotinamide adenine dinucleotide (NADH) [9].

CUVAF has demonstrated associations with both clinical and subclinical signs of sun damage [10, 11]. Studies have shown that individuals with pterygium exhibit significantly greater CUVAF areas than those without. McKnight et al. found that every 10 mm^2^ increase in CUVAF area increased the odds of having a pterygium by 23% [12]. Kumar et al. [13] reported over a 200-fold increase in risk for those in the highest quartile of CUVAF area. Interestingly, autofluorescence is sometimes present even in individuals without clinically visible lesions, suggesting it may serve as a marker of early or preclinical ocular surface damage. This subclinical CUVAF often manifests in nasal and temporal conjunctival regions, reflecting geographic patterns of solar exposure and eyelid shielding [14]. Population-based studies have further highlighted considerable variability in CUVAF, influenced by cumulative UV exposure1, genetic predisposition [14], seasonality [15], age of onset and protective behaviours such as sunglass wear [16, 17]. However, population-level reference ranges remain poorly characterised, reinforcing the need for normative data and standardised imaging protocols [14, 18]. Another application for this technology is in myopia research. We have demonstrated an inverse relationship of CUVAF and myopia in young adults [12, 19].

The standard technique for CUVAF imaging employs a benchtop digital single-lens reflex (DSLR) camera equipped with a macro lens and external UV-filtered flash, mounted on a table with head and chin rests to stabilize the subject. Photographs are taken of the nasal and temporal conjunctiva in a darkened room to enhance autofluorescence contrast. While effective, this setup is costly, bulky, and generally confined to research institutions and specialised clinics, limiting its accessibility in primary care or community settings.

To address these limitations, alternative imaging solutions are being explored. Kumar et al. [13] adapted the DSLR-based technique using a smartphone camera fitted with a UV transmission filter to measure CUVAF in a South Indian cohort. This approach offers a promising avenue for expanding access, especially in under-resourced areas, but it has not yet been formally validated.

This study aims to validate a smartphone-based clip-on device for CUVAF imaging by comparing its performance against the gold-standard DSLR system. Specifically, we evaluate the agreement in CUVAF area and intensity measurements between the two systems. By establishing the reliability and accessibility of this portable tool, we aim to support its adoption in diverse clinical and community settings.

## Methods

A prospective cross-sectional, repeated-measures design was used to compare the detection and quantification of conjunctival ultraviolet autofluorescence (CUVAF) using a smartphone-based imaging system versus a DSLR-based system. The use of repeated measures within the same participant controlled for interindividual variability in CUVAF location, area, and intensity. DSLR images served as the gold-standard reference for comparative validation.

Participants (*n* = 95, 41 males) were recruited from co-author MC’s pterygium clinic in Sydney. Inclusion criteria were: (1) age ≥ 18 years; (2) clinical diagnosis of a sun-related ocular surface disorder on slit-lamp examination; and (3) consent to participate.

### CUVAF Imaging

**Fig. 1:**
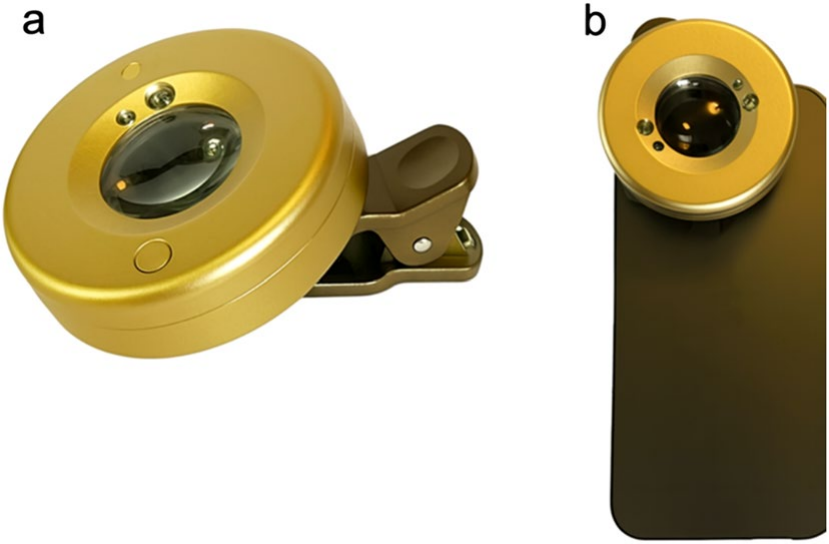
Smartphone-based imaging system for CUVAF. **a** UV transmission filter light source attachment. **b** Attachment mounted on an iPhone 13
- *DSLR-Based Gold-Standard System*
  The reference system comprised a Nikon D100 DSLR camera (Nikon Corp., Tokyo, Japan) with a 105 mm macro lens and dual Metz 36 C-2 external flashes equipped with crescent-shaped UV filters. The excitation source had similar spectral properties (300–400 nm, peak 365 nm). A B + W 62 420 UV cut filter (Schneider-Kreuznach, Germany) blocked UV light from reaching the detector, enabling only autofluorescent signals to be recorded. The DSLR was mounted on a slit-lamp for stabilisation and consistent alignment. The system was mounted on a slit-lamp for image stabilisation (Fig. 2).

- *Smartphone-Based System*
  The portable CUVAF system used an iPhone 13 (Apple Inc., Cupertino, CA, USA) equipped with a custom-designed clip-on attachment developed in-house for this study. The design was inspired by the Photobluminator (Eidolon, MA, USA), with significant modifications made to accommodate the optical components used in this study.
  The clip-on housed an ultraviolet (UV) transmission LED light source with a peak wavelength of 365 nm and an emission range of 300–400 nm, selected to match the DSLR-based gold-standard system. Although the emission spectrum spanned 300–400 nm, the peak intensity was at 365 nm, corresponding to the UVA range. Emission in the UVB region (280–315 nm) was minimal and unlikely to contribute significantly to excitation. The brief duration of UV exposure (< 2 s per image) and low-power LED output were consistent with previously published CUVAF imaging protocols and considered safe for ocular tissue. The light source emitted a divergent beam and was positioned adjacent to the camera lens. The control visible light source was a standard white LED (400–700 nm), mounted identically to the UV LED, used to capture baseline conjunctival images without autofluorescence for comparison.
  An off-the-shelf AR-IR cut filter (Quanmin 6.5 mm Optical AR-IR Cut Filter) was used to minimise infrared and ambient light interference. The AR-IR cut filter is a low-pass optical-grade glass filter with ≥ 95% transmission in the 400–620 nm range. Additionally, a 7.5× macroscopic lens was integrated into the clip-on design to allow high-resolution imaging of the conjunctiva at close range. Polarisation was not specifically controlled for, consistent with prior CUVAF imaging protocols.
  The phone was handheld and aligned at a consistent distance of ~ 3 cm from the participant’s eye, using real-time visual confirmation via the screen and verbal guidance for gaze direction. Alignment was guided to ensure consistent exposure of the conjunctiva and sclera. For participants prone to blinking or movement, a short video was recorded and a high-quality still frame was extracted. Imaging was conducted in a darkened room to reduce ambient light interference. Control images using visible light only were obtained using a white LED mounted on the same clip-on (Fig. 1).

**Fig. 2:**
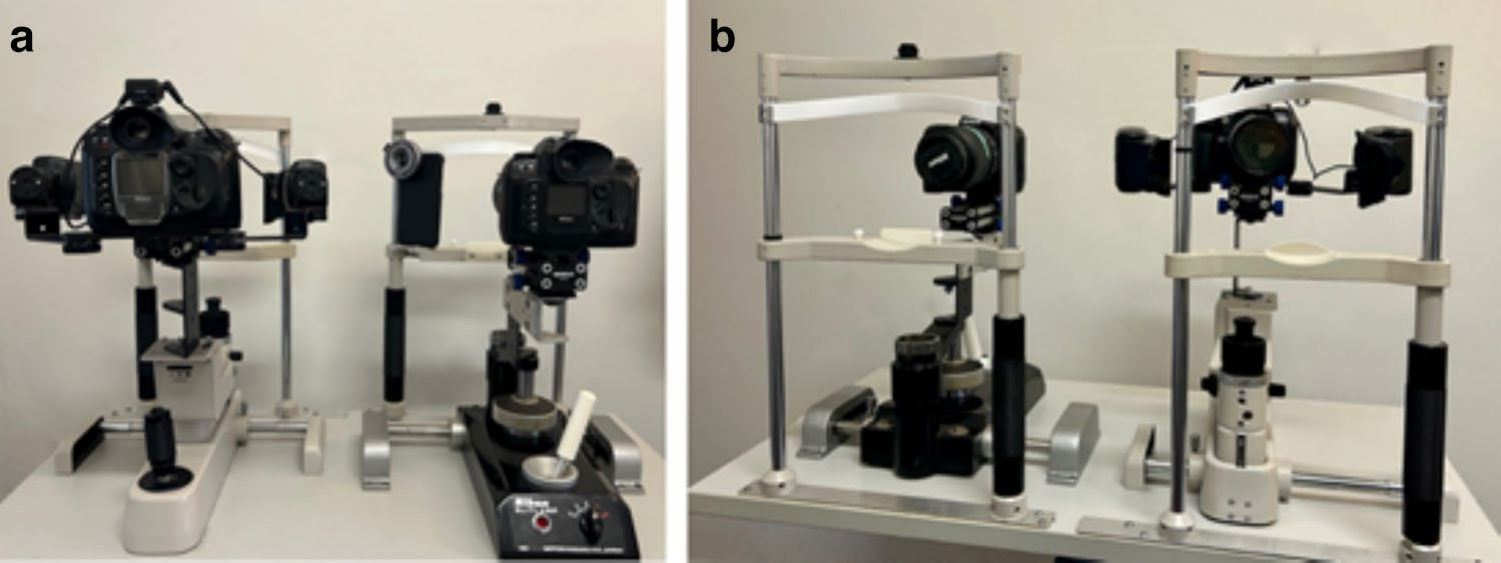
DSLR-based imaging systems. **a** Researcher perspective: Left—CUVAF; Right—control. **b** Patient perspective: Left—control; Right—CUVAF
  For each eye, nasal, temporal, and anterior views were obtained using both systems, resulting in 12 images per participant. DSLR visible light control images used a standard lens and visible light filter mounted on the same slit-lamp.
  All images were saved in JPEG format. Smartphone images had a resolution of 4032 × 3024 pixels at 72 ppi, while DSLR images had a resolution of 3008 × 2000 pixels at 300 ppi. Both were deidentified before analysis.

### CUVAF Region Quantification

**Fig. 3:**
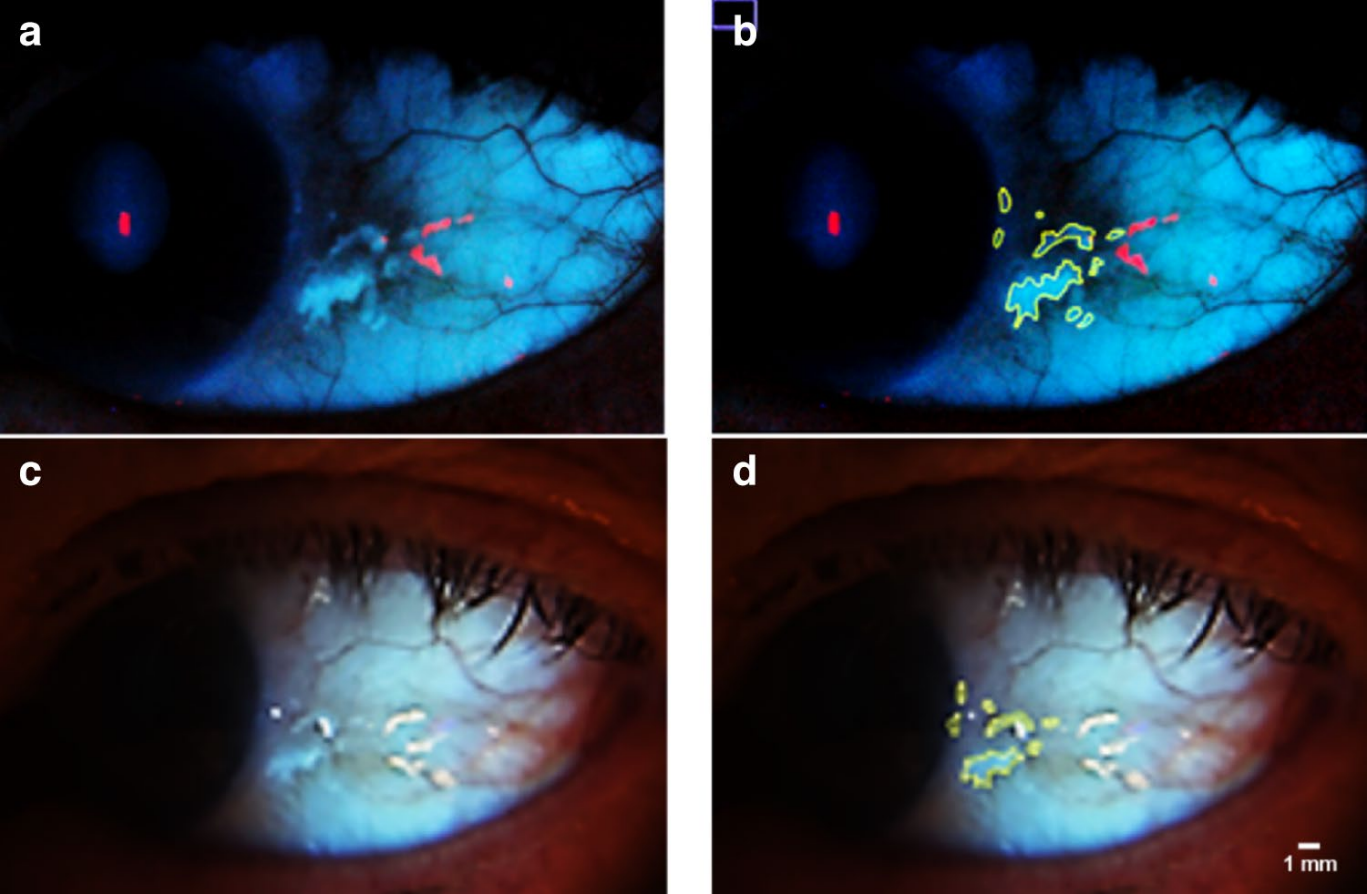
Annotated CUVAF regions. **a**, **b** DSLR image and annotated ROI. **c**, **d** Smartphone image and annotated ROI

The assessor was blinded to participants’ medical condition status. DSLR images were analysed before smartphone images to minimise bias. ImageJ software (NIH, USA) was used to manually delineate CUVAF regions. Using the “freehand selection” tool, CUVAF regions were saved as regions of interest (ROIs), and the “zoom” tool enabled detailed inspection. To reduce inter-image variability, all measurements were obtained under standardised brightness and contrast settings. Fig. 3 shows the annotated CUVAF regions in matched smartphone and DSLR images.

The “measure” tool recorded the total area and mean pixel intensity of each ROI. The “histogram” function produced intensity frequency distributions, and binary masks were created using the “mask” tool for potential future AI analysis (Fig. 3).

CUVAF area was calibrated against the visible sclera, which was manually traced as a secondary ROI. This approach was used to account for intra-individual differences in gaze angle and image framing, which affect the amount of visible scleral area. Equation 1 defines the calculated “area ratio”:

1 $$\text{Area ratio}= \frac{\text{Area of CUVAF region}}{\text{Area of visible scleral region}}$$

To compute the “intensity ratio,” the CUVAF ROI was duplicated and placed over an adjacent undamaged scleral region of equivalent size and shape. Equation 2 defines the “intensity ratio”:

2 $$\text{Intensity ratio}= \frac{\text{Mean pixel intensity of CUVAF region }}{\text{Mean pixel intensity of background region}}$$

All data, including raw scleral and CUVAF area values (in pixels), were recorded in Microsoft Excel (Microsoft Corp., Redmond, WA, USA).

### Statistical Analysis

All analyses were conducted using GraphPad Prism (GraphPad Software, Boston, MA, USA). To compare CUVAF area ratios and intensity ratios between DSLR and smartphone images, Wilcoxon signed-rank tests were used. Agreement was visualised using Bland–Altman plots, comparing device-specific measurements and their differences. Sample size was based on feasibility and comparable prior validation studies using CUVAF imaging. A formal power calculation was not conducted, but the sample of 95 participants exceeds that of previous work assessing UV-related ocular biomarkers and provides adequate statistical power to detect moderate differences and agreement patterns between methods.

## Results

### Participant Characteristics

A total of 95 participants were included in the study. The mean age was 58 ± 14 years, and 42% were male. Table 1 summarises participant demographics and clinical characteristics.

**Table 1:** Participant demographics and clinical characteristics

Characteristic	Description
Age (years)—mean ± SD	58 ± 14
Sex—*n*	
Male	41
Female	54
Ethnicity—*n*	
Caucasian	83
Hispanic	1
Southeast Asian	5
Other	6
Height (m)—mean ± SD	1.7 ± 0.11
Weight (kg)—mean ± SD	71.4 ± 13.3
BMI—mean ± SD	24.3 ± 2.5
Eye conditions—*n*	
Pterygium	52
Dry eye disease	7
Red eyes	3
Cataract	3
Pinguecula	7
Other	14
Unspecified	9

### Image Acquisition

Of the 95 participants recruited, 15 (16%) exhibited no detectable CUVAF signal on either imaging system and were therefore excluded from the comparative analysis, as no lesions were available for device comparison. An additional 7 participants were excluded due to image artefacts or poor focus. For the remaining 73 participants, a total of 876 images were successfully acquired from both eyes using both imaging systems and included in the final analysis. (Supplementary File A presents a selection of side-by-side images captured with the two devices).

### Comparison of CUVAF Features

The mean normalised CUVAF area (i.e., CUVAF/total sclera area) was higher in smartphone images (0.034 ± 0.03) compared to DSLR images (0.025 ± 0.02, *p* < 0.01). The median area was similarly greater for smartphone (0.02) versus DSLR (0.018). The 95% confidence interval for smartphone area ranged from 0.027 to 0.041, while that for DSLR ranged from 0.02 to 0.031.

Normalised CUVAF intensity values (i.e., CUVAF/adjacent background intensity) were slightly higher on average for DSLR imaging (1.44 ± 0.33) than for smartphone imaging (1.43 ± 0.29, *p* = 0.2). Median values and interquartile ranges are provided in Table 2.

**Table 2:** Descriptive statistics for normalised CUVAF area and intensity

CUVAF Metric	Device	Mean	Median	SEM	SD	95% CI
Area	DSLR	0.025	0.02	0.002	0.02	[0.02, 0.031]
	Smartphone	0.034	0.03	0.003	0.03	[0.027, 0.041]
Intensity	DSLR	1.44	1.34	0.038	0.33	[1.37, 1.52]
	Smartphone	1.43	1.25	0.036	0.29	[1.35, 1.5]

Statistical comparisons were performed using paired non-parametric tests. The Wilcoxon signed-rank test demonstrated a statistically significant difference in normalised CUVAF area between DSLR and smartphone (*p* < 0.01). However, the difference in normalised intensity between DSLR and smartphone was not statistically significant (*p* = 0.2).

### Bland-Altman Analysis

**Fig. 4:**
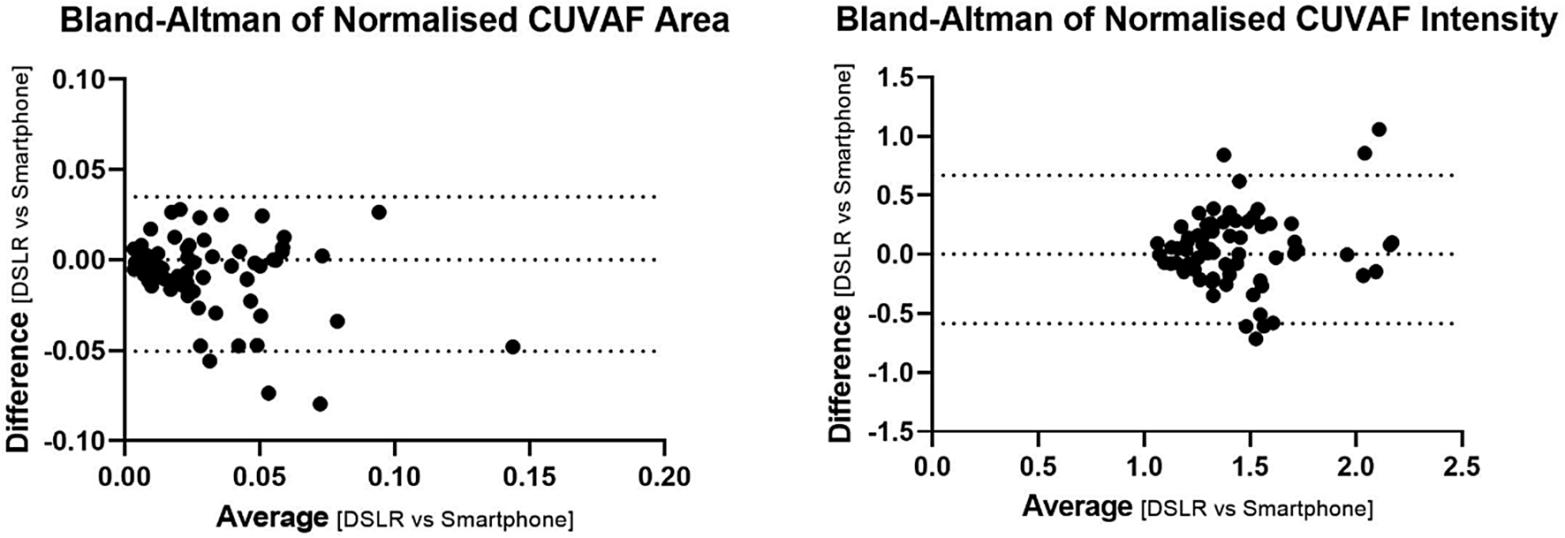
Bland–Altman plots comparing normalised CUVAF area (left) and intensity (right) between DSLR and smartphone measurements. Upper and lower dashed lines indicate 95% limits of agreement. Each point represents a patient-level difference vs. average

To assess agreement between DSLR and smartphone measurements, Bland–Altman analysis was performed. For normalised area, the mean difference was − 0.007 (SD = 0.02), with 95% limits of agreement ranging from − 0.05 to 0.034. For normalised intensity, the mean difference was 0.041 (SD = 0.31), with limits of agreement from − 0.58 to 0.66 (Fig. 4).

## Discussion

This study aimed to compare the quantification of conjunctival ultraviolet autofluorescence (CUVAF) area and intensity between images captured using a smartphone-based device and the gold-standard DSLR-based system. We observed that while the smartphone-based device produced significantly larger CUVAF area measurements, the difference is unlikely to be clinically meaningful. In contrast, intensity measurements were statistically indistinguishable between the two devices. These findings suggest that smartphone-based CUVAF provides a viable and accessible method for quantifying ocular UV damage. The paired, repeated measures design of this study allowed direct comparison between imaging modalities without confounding from inter-individual differences in UV exposure or lesion characteristics. Overall, our results support the utility of the smartphone-based system as a portable and cost-effective alternative to the benchtop DSLR-based system, particularly in contexts requiring accessibility and ease of use [7, 13].

Smartphone images yielded significantly higher normalised area ratios than DSLR images. Despite this statistical difference, the Bland-Altman analysis indicated a modest bias (− 0.007) with 95% limits of agreement well within a clinically acceptable range. Moreover, the two devices showed complete agreement when classifying CUVAF lesions as either present or absent, a distinction that Sherwin et al. [20] suggest may be more clinically relevant than precise area measurements. The larger area measurements from smartphone images may reflect factors unrelated to disease or damage severity. For instance, minor variations in the angle of image capture, distance from the eye, and gaze direction (particularly in handheld, non-slit-lamp-based imaging) can affect the amount of visible sclera and thereby alter the denominator used in normalised area calculations. Additionally, differences in underexposure between DSLR and smartphone images may have obscured scleral boundaries and influenced manual region tracing. While the use of the scleral area as a calibration reference improved practicality, it introduces variability that could be minimised in future studies using standardised ruler calibration or fixed gaze alignment tools such as LED diodes [7, 21].

Another explanation for the proportional bias is that larger CUVAF regions are inherently more variable due to irregular shapes and operator-dependent tracing. Similar trends have been described in earlier studies, where greater variation in area measurement occurred as lesion size increased [20, 22]. Despite these methodological differences, the consistency in lesion classification and the relatively small average discrepancy in area support the smartphone device’s adequacy for clinical use.

In contrast to area, CUVAF intensity ratios did not differ significantly between devices. The mean difference was small, and the Bland–Altman analysis revealed symmetrical limits of agreement, suggesting high reliability. This likely reflects the fact that intensity measurements are less sensitive to image framing, gaze direction, and minor exposure differences. While some scatter and outliers were observed, potentially due to ambiguous lesion boundaries or bright artefacts from reflections, these limitations are not unique to smartphone imaging and have been reported in DSLR-based studies [21]. The absence of a clear intensity border for autofluorescent regions makes precise delineation challenging and may explain minor inconsistencies. Nonetheless, the strong agreement in intensity suggests that smartphone imaging is well-suited for quantifying UV exposure, particularly when tracking changes over time or comparing populations [11, 23].

Our study had several limitations. First, although the smartphone-based device yielded a statistically significant difference in CUVAF area ratios compared to the DSLR system, this discrepancy appears minor in clinical terms and likely arises from image acquisition factors such as viewing angle and gaze direction, rather than any intrinsic limitations of the device. Considering that many studies prioritise the binary presence or absence of CUVAF rather than precise area, and recognising inherent variability even in DSLR-based measurements [12, 20, 24], our findings still support the smartphone system’s promise.

Second, manual delineation of CUVAF regions remains subjective, introducing variability in both area and intensity quantification. Although masking and sequential image analysis were employed to minimise bias, more automated segmentation tools could enhance consistency and reproducibility [16]. Differences in resolution, field of view, and image exposure across devices may have also introduced minor inconsistencies, despite efforts to standardise procedures. Additionally, differences in optical filter characteristics likely contributed to variations in background fluorescence between systems: the smartphone-based tool employed an off-the-shelf AR-IR Cut Filter, which may permit some excitation light leakage and contribute to residual background glow. In contrast, the DSLR system used a B + W 62 420 UV cut filter, which more effectively blocks reflected excitation light, resulting in crisper, less glare-prone images.

Finally, while the sample size (*n* = 73 for comparative analysis) was sufficient for the primary aim, the study cohort was predominantly Caucasian, limiting generalisability. Prior studies suggest that pigmentation characteristics, such as hair and eye colour, may influence CUVAF expression more than skin tone alone [10]. Future studies should include more ethnically diverse populations to ensure broader applicability.

To further validate the clinical utility of smartphone-based CUVAF imaging, future studies could incorporate gaze fixation tools (e.g., LED diodes) and calibrate area measurements using fixed physical references such as ruler scales. Longitudinal research assessing the reliability of smartphone imaging for tracking CUVAF progression over time would also provide valuable insights, particularly for patient monitoring. Integration of AI-powered segmentation algorithms could reduce subjectivity in CUVAF quantification and enable rapid, large-scale screening applications.

Pterygium is quintessentially a UV-related disease and its presence predates and is an indicator of increased risk of both keratinocyte cancer [25] and cutaneous melanoma [26]. Since we have demonstrated a relationship between CUVAF and pterygium prevalence [27, 28], CUVAF may be an predictor of future skin cancer risk. Consequently, a readily accessible ocular surface biomarker of both ocular and systemic UV damage may prove to be a useful tool in managing health risks of excessive UV exposure.

In conclusion, a smartphone-based CUVAF imaging device provides reliable and clinically meaningful quantification of UV-induced conjunctival autofluorescence. Although area ratios were slightly higher with smartphone imaging, this difference is likely not clinically significant. The smartphone system offers a cost-effective, portable, and practical alternative to the DSLR-based gold-standard, supporting broader accessibility to CUVAF imaging in clinical, research, and educational settings.

## Supplementary Information

Below is the link to the electronic supplementary material.

Supplementary file1 (PDF 420 kb)
