# Supplementary material for: Validation of a Portable Tool for Conjunctival Ultraviolet Autofluorescence Imaging
Source: Ann Biomed Eng. 2026 Jan 28;54(6):1738–45. doi: 10.1007/s10439-026-03992-3 (PMC13186899; doi:10.1007/s10439-026-03992-3)
Supplement: Supplementary file 1 — Supplementary file1 (PDF 420 kb) [file 10439_2026_3992_MOESM1_ESM.pdf]

Supplementary file A

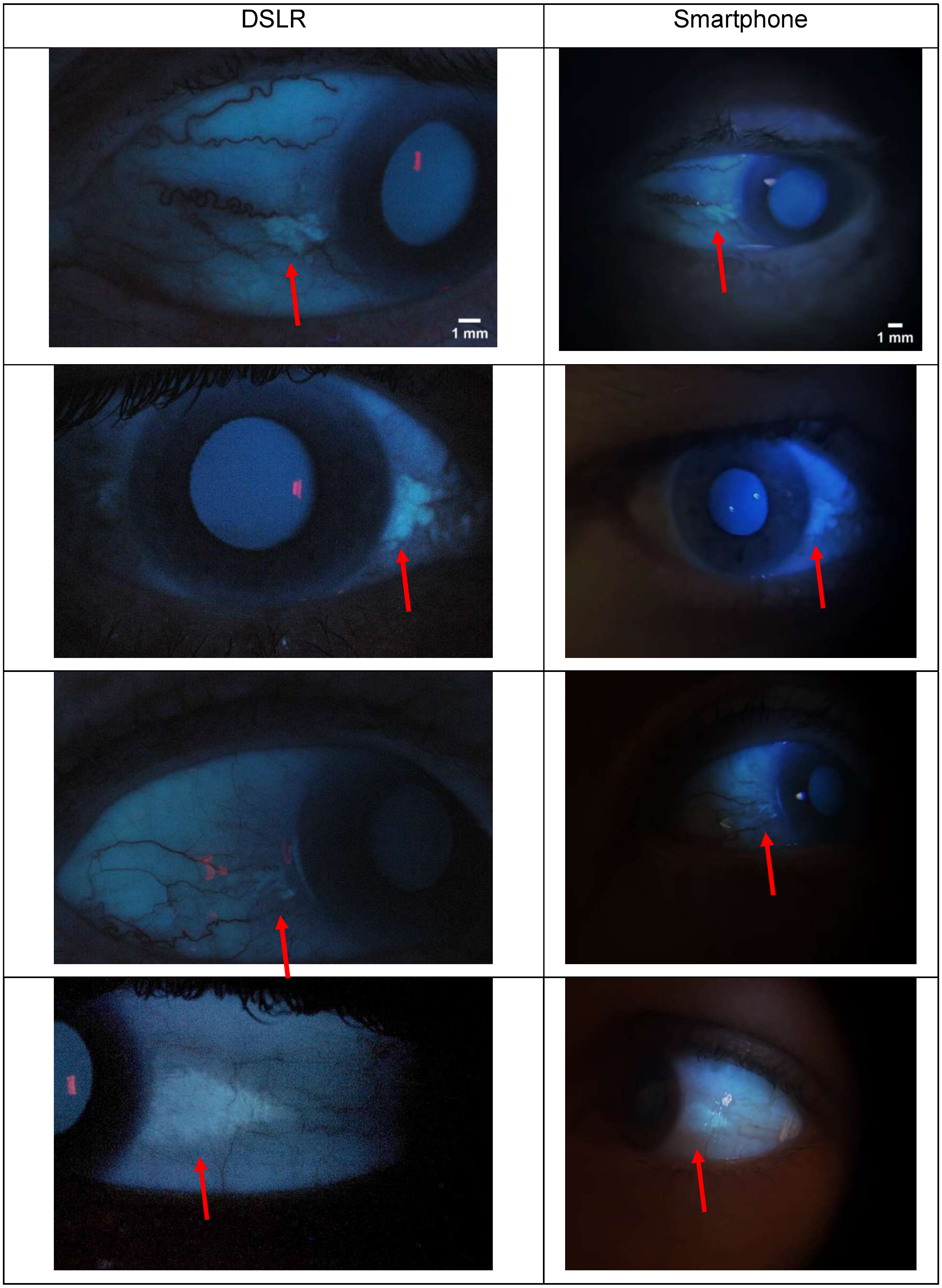

Figure S1. Representative side-by-side CUVAF images captured using the DSLR and smartphone-based devices.
